# Effects of Drinking Oxygenated Water on Blood Oxygen Saturation During Exercise Under Normobaric Hypoxic Conditions: A Randomized Placebo-controlled Single-blinded Trial

**DOI:** 10.14789/jmj.JMJ21-0052-OA

**Published:** 2022-06-02

**Authors:** HIDENORI IZAWA, MASASHI NAGAO, SHOJIRO NOZU, YUKI SHIOTA, KOSUKE FUKAO, HIROFUMI NISHIO, YUJI TAKAZAWA

**Affiliations:** 1 Graduate School of Health and Sports Science, Juntendo University Chiba, Japan; 1 Graduate School of Health and Sports Science, Juntendo University Chiba, Japan; 2 Department of Sports Medicine, Juntendo University, Chiba, Japan; 2 Department of Sports Medicine, Juntendo University, Chiba, Japan; 3 Innovative Medical Technology Research & Development Center, Juntendo University, Tokyo, Japan; 3 Innovative Medical Technology Research & Development Center, Juntendo University, Tokyo, Japan; 4 Institute of Health and Sports Science & Medicine, Juntendo University, Chiba, Japan; 4 Institute of Health and Sports Science & Medicine, Juntendo University, Chiba, Japan

**Keywords:** water, oxygen, hypoxia, randomized controlled trial

## Abstract

**Objectives:**

This study aimed to investigate the effects of drinking oxygenated water on oxygen saturation during exercise under normobaric hypoxic conditions.

**Materials:**

A randomized placebo-controlled single-blinded trial was performed. Twenty-two healthy adults (16 men and 6 women), with a mean age (standard deviation) of 22.4 (2.73) years, participated in the study. The participants were randomly assigned to one of two groups: an OX group (drinking oxygenated mineral water) and a control group (drinking normal mineral water). Both groups performed walking exercises under normobaric hypoxic conditions. Blood oxygen saturation (SpO_2_), pulse rate (PR), and walking distance were measured during exercise.

**Results:**

SpO_2_ decreased and PR increased during exercise in both groups. The decrease in SpO_2_ was smaller and the increase in PR was greater in the OX group compared with those in the control group. No significant difference was found in walking distance between the two groups.

**Conclusions:**

Drinking oxygenated water before exercise may inhibit SpO_2_ reduction under normobaric hypoxic conditions.

## Introduction

Enhancement of athletic ability is of interest not only for athletes, but for anyone engaged in playing sports. In recent years, to exploit the beneficial effects of oxygen on athletic performance, it has been proposed that drinking water containing high concentrations of dissolved oxygen may have the potential to improve athletic performance. A previous study demonstrated that high concentrations of dissolved oxygen in water (up to 40 times that of typical drinking water) improved athletic performance^1)^. In addition, oxygenated water is marketed as a drink that can replenish water and oxygen, with claimed benefits including aiding recovery from fatigue after exercise and increasing concentration. However, scientific evidence regarding the effects of high-concentration oxygenated water remains controversial^2-8)^. Because excessive enhancement of athletic performance can be detrimental to an athlete’s health and interfere with fair competition, well-designed scientific experiments are needed.

Under normal conditions, the partial pressure of oxygen is known to decrease with high-intensity training^9, 10)^. In a study investigating exercise under normobaric hypoxic conditions, peripheral blood oxygen saturation (SpO_2_) was reported to be significantly decreased with bicycle training in healthy adults^11)^. On the basis of these findings, we hypothesized that the intake of high-concentration oxygenated water before and during exercise could prevent SpO_2_ reduction during exercise in a hypoxic environment.

Therefore, the current study aimed to evaluate changes in blood oxygen saturation during exercise under hypoxic conditions after consumption of high-concentration oxygenated water in normal adults.

## Materials and Methods

This randomized placebo-controlled single-blinded trial was performed from July to August 2021. The current study was approved by the institutional human ethics committee (2021-77) at Juntendo University School of Health and Sports Science, and was in compliance with the Declaration of Helsinki and existing legal regulations. Written consent was obtained from all participants before their enrollment. The inclusion criteria were as follows: healthy men or women aged > 20 years. The exclusion criteria were as follows: body temperature > 37.5 °C; SpO_2_ < 95%; systolic blood pressure > 145 mmHg or diastolic blood pressure < 90 mmHg; history of heart or pulmonary disease; and other comorbidities, disorders and diseases that could affect the results. We recruited 22 participants, who were randomly assigned to either the control group (drinking normal mineral water) or the OX group (drinking oxygenated mineral water with high oxygen concentration). Participants were blinded from group allocation. Age, sex, body height, body weight, smoking history, and sports habits were collected as demographic data.

## Intervention

**Figure 1:**
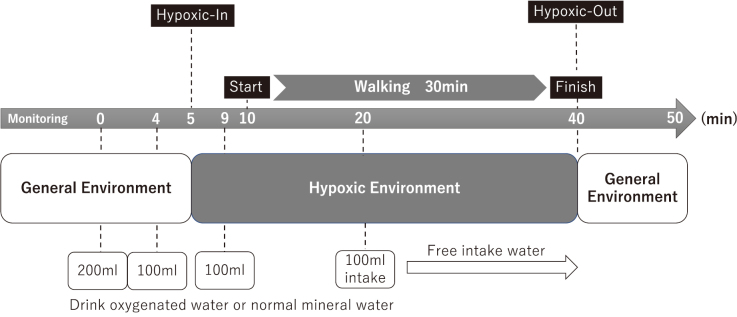
Flow of the experiments Intake: drinking oxygenated water or normal mineral water

A bottle of normal mineral water (control group) or oxygenated mineral water (OX group) was given to participants before the experiment began. An OXMAX (WellsO_2_ Inc, Tokyo, Japan) oxygen capsule was used to make the oxygenated water. The same commercially available bottled mineral water at room temperature was used for the experiments in both groups. The experiment was conducted at the High-Alti Training Studio (High Altitude Management Co., Ltd, Tokyo, Japan), where a normobaric hypoxic environment (oxygen partial pressure of 15.2%, equivalent to 2600 m above sea level) was available. During the experiment, participants were asked to walk for 30 min on a self-propelled treadmill (Speedboard ProXL, Speed Fit, USA) under normobaric hypoxic conditions, and during exercise, SpO_2_ and pulse rate (PR) were measured every 2 s using a pulse oximeter (RingO_2_, Viatom Technology Co. Ltd). Exercise intensity was set at 11-13 on the subjective fatigue Borg scale^12)^, and the score was recorded every 3 min during exercise. The participants were instructed to drink 200 mL of water 10 min before walking, 100 mL 6 min before walking, 100 mL 1 min before walking, and 100 mL 10 min after starting walking. Participants entered the hypoxic room 5 min before the exercise and left the room after 30 min of walking exercise. After the exercise period, participants rested for 10 min in a normoxic environment (Figure 1).

## Statistical analysis

Before the experiments, the average SpO_2_ and PR values under the normal environment were used as baseline values. These were analyzed every 5 min. The mean values for each phase were compared between the two groups. In addition, the walking distance displayed on the treadmill monitor at the end of the exercise period was recorded and compared between the two groups. We used two-way analyses of variance for comparing the mean quantitative variable changes. A p-value less than 0.05 was considered to indicate statistical significance. Statistical analyses were performed using GraphPad Prism 9 (GraphPad Software Inc., La Jolla, CA, USA).

## Results

Sixteen men and six women participated in this study. The mean age (standard deviation; SD) was 22.4 (2.73) years. The mean height (SD) was 167.1 cm (8.0) and mean weight (SD) was 63.5 kg (15.3), as shown in Table 1. Of the 22 participants, 3 were smokers. The participants, on average, exercised for 2.2 (2.15) days per week. The resting SpO_2_ values (95% confidence interval [CI]) in both the control and OX groups were 97.2% (1.49) and 95.0% (3.98), respectively, and no significant difference was observed (P = 0.13).

**Table 1:** Demographic characteristics of participants

	Control (SD) n=11	Oxgen Water (SD) n=11	P-value
Sex (Male/Fmale)	8/3	8/3	
Age (Y)	22.7 (2.53)	22.1 (3.02)	0.60
Height (㎝)	167.7 (8.94)	166.6 (7.36)	0.76
Weight (㎏)	66.7 (18.77)	60.4 (10.85)	0.34
Smoker	3/11	0/11	
Exercise habits (days/week)	1.5 (2.34)	2.9 (1.81)	0.14
SpO_2_ (%)	97.2 (1.49)	95.0 (3.98)	0.13
Pulse rate (bpm)	85.1 (10.65)	79.7 (18.47)	0.41

The walking distances during exercise in the OX (SD) and control groups (SD) were 3.5 (0.3) km and 3.39 (0.27) km, respectively, demonstrating no significant difference (P = 0.32). During walking, the Borg scores (SD) in the OX and control groups were 12.0 (0.7) and 11.5 (0.8), respectively, showing no significant difference (P = 0.14).

**Figure 2:**
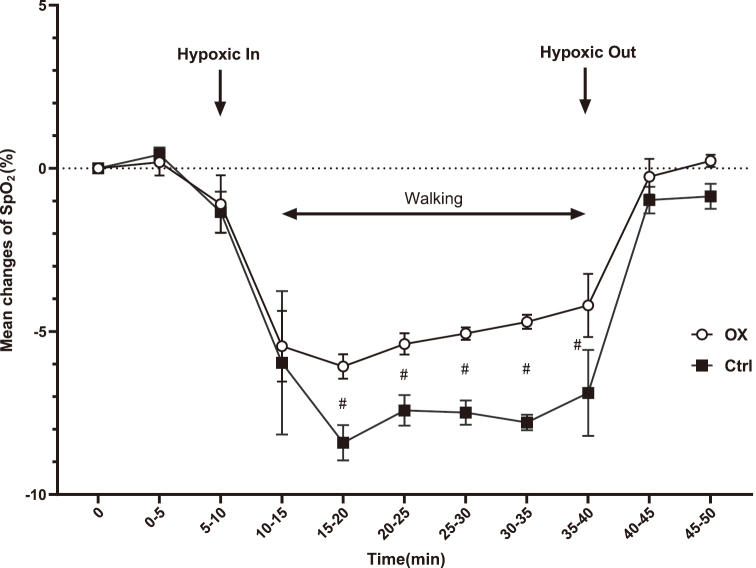
Changes in SpO_2_ during 30 min walking exercise Error bar, 95% confidence interval. #, P < 0.05 OX: OX group (drinking oxygenated mineral water with high oxygen concentration) Ctrl: Control group (drinking normal mineral water)

**Figure 3:**
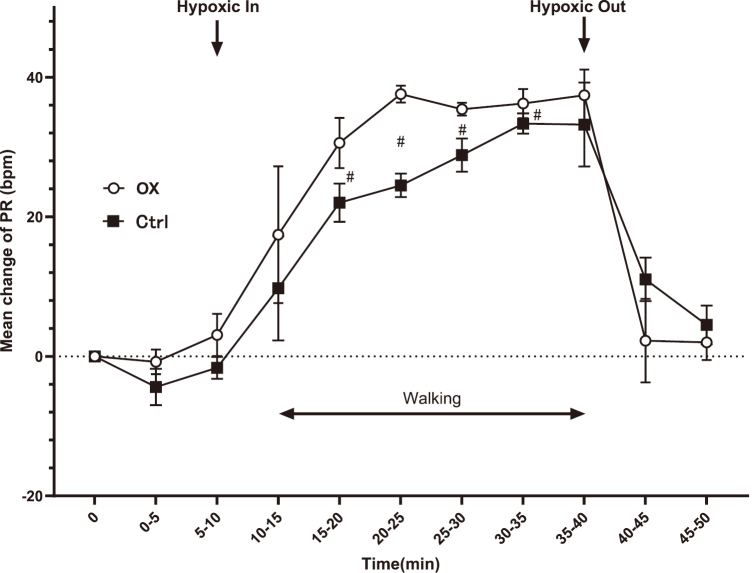
Changes in pulse rate (PR) during 30-min walking exercise Error bar, 95% confidence interval. #, P < 0.05 PR: pulse rate OX: OX group (drinking oxygenated mineral water with high oxygen concentration) Ctrl: Control group (drinking normal mineral water)

At 10-40 min, SpO_2_ reduction was observed in both groups compared with the baseline values (P < 0.05). Differences between the two groups were observed at 15-40 min (Figure 2). Regarding PR, the baseline values of the control and OX groups (95% CI) were 85.1 (10.7) and 79.7 (18.5) bpm, respectively. During exercise, the PR values at 10-40 min in both groups were increased compared with the baseline values. Furthermore, at 10-35 min, the PR values in the OX group were increased compared with those in the control group (P < 0.05), similar to SpO_2_ (Figure 3).

## Discussion

In this study, we compared temporal changes in SpO_2_ and PR during exercise under hypoxic conditions after consumption of oxygenated mineral water or normal mineral water. The results demonstrated a decrease in SpO_2_ and an increase in PR during walking exercise in both groups under hypoxic conditions. Moreover, the decrease in SpO_2_ was smaller and the increase in PR was greater in the group that drank oxygenated water compared with the group that drank normal mineral water.

A decrease in arterial partial oxygen pressure (PaO_2_) and arterial oxygen saturation (SaO_2_) caused by exercise is known as exercise-induced arterial hypoxemia (EIAH)^13)^. EIAH is defined as a decrease in PaO_2_ of ≥ 10 mmHg or a decrease in SaO_2_ of ≥ 3% compared with these values at rest. SpO_2_ and transcutaneous measurement of SaO_2_ are almost identical^14)^. In this study, SpO_2_ was decreased by ≥ 3% in both groups, indicating that the participants exhibited EIAH. A previous study reported that maximal performance capacity is impaired in highly trained cyclists working under 87% SaO_2_, but not under a milder desaturation level of 90%^15)^. Arterial desaturation occurs in healthy, highly trained endurance athletes during heavy exercise, and the level of arterial desaturation is inversely related to maximal oxygen consumption (VO_2_max)^10)^. Although drinking oxygenated water did not protect against EIAH in the present study, SpO_2_ was maintained at 90%. Given that exhaustion caused by EIAH shortens the exercise time and decreased SaO_2_ affects muscle fatigue^16)^, drinking oxygenated water may protect athletes from performance decreases.

Environments with low SpO_2_ include high-altitude situations, such as in-flight or alpine environments. SpO_2_ decreases during exercise in a hypoxic environment and results in a lack of oxygen supply to tissues, leading to illness or fatigue^17)^. Reports on mountaineering exercise show that SpO_2_ decreases and PR increases, particularly when physical conditions are not good. Understanding the hypoxic state of the body and managing and assessing its physical condition are effective for preventing acute mountain sickness^14, 18)^. Reducing the decrease in SpO_2_ by drinking oxygenated water may prevent acute mountain sickness, prolong walking time, and increase walking speed. Similarly, in in-flight environments (altitude of 40,000 ft), the oxygen concentration is approximately 16%, and SpO_2_ is reported to decrease in individuals with respiratory dysfunction^19)^. Even in healthy individuals, SpO_2_ decreases significantly in in-flight environments^20)^. Therefore, drinking oxygenated water before or during flight may suppress SpO_2_ reduction and help maintain physical condition.

Oxygen is mainly absorbed into the body by inhalation. However, a previous study in pigs demonstrated that administering oxygenated water into the stomach increased SaO_2_. These results suggest that the administration of oxygenated water could be an alternative route of oxygen absorption^21)^. In the present study, oxygenated water with an oxygen concentration of 110 ppm (110 mg O_2_/L) was used, but the speed of absorption into the body was unclear. In a previous study^22)^, different concentrations of oxygenated water (40, 80, and 150 mg O_2_/L) were intraperitoneally injected in rabbits, and the authors demonstrated that different oxygen concentrations have different absorption speeds. Optimizing oxygen levels in water could potentially lead to better outcomes, and further research on this topic is warranted.

Our data demonstrated that PR was higher in the OX group compared with the control group. We hypothesized that SpO_2_ decreases in hypoxic environments, as demonstrated in a previous study^23)^. A previous study involving a walking exercise experiment reported that pulse oximeter measurements in the hand may provide false readings because of body movement, and this effect is more likely to be seen in the measurement of PR compared with that of SpO_2_^24)^. Alternatively, it is possible that our findings were caused by differences in the physical abilities of the participants. Although participants were randomly assigned and their baseline demographic data were similar, some of the participants in the OX group may have had inferior physical abilities. However, we monitored exercise intensity using the Borg scale^12, 25)^, and walking distances did not differ between groups. Although it is unclear why participants in the OX group had higher PR, it is important to be aware of this phenomenon to identify individuals who might experience harmful effects of increased PR.

One strength of the current study is the experimental design involving a randomized placebo-controlled single-blinded trial, which would be expected to reduce the effects of bias. However, this study also involved several limitations. First, the sample size was small. Although a small sample size can result in type 2 error, it is unlikely that this error occurred in the current study because the differences between the groups were statistically significant. Second, the participant selection method may have affected the results. We enrolled young adults aged 20-29 years. Although the changes in younger and older populations were not investigated, changes in SpO_2_ and PR may be more prominent in older participants. Third, the effects of oxygenated water under normal conditions remain unclear. In conclusion, the current study demonstrated that SpO_2_ was decreased and PR was increased under normobaric hypoxic conditions. Moreover, consumption of oxygenated water containing 110 ppm oxygen during walking exercise under normobaric hypoxic conditions suppressed SpO_2_ reduction.

## Funding

This study was funded by WellsO_2_ Inc, Tokyo, Japan.

## Author contributions

MN and SN analyzed and interpreted the data of the participants in this study. All authors read and approved the final manuscript.

## Conflicts of interest statement

WellsO_2_ Inc funded this study. However, none of the authors were employees of the company nor received any honoraria.
